# Influences of circulatory factors on intervertebral disc aging phenotype

**DOI:** 10.18632/aging.103421

**Published:** 2020-06-11

**Authors:** Changbin Lei, Debora Colangelo, Prashanti Patil, Vivian Li, Kevin Ngo, Dong Wang, Qing Dong, Matthew J. Yousefzadeh, Hongsheng Lin, Joon Lee, James Kang, Gwendolyn Sowa, Tony Wyss-Coray, Laura J. Niedernhofer, Paul D. Robbins, Derek M. Huffman, Nam Vo

**Affiliations:** 1 Department of Orthopaedic Surgery, The First Affiliated Hospital of Jinan University, Guangdong 510630, China; 2 Ferguson Laboratory for Orthopaedic Research, Department of Orthopaedic Surgery, University of Pittsburgh, E1641 Biomedical Science Tower, Pittsburgh, PA 15213, USA; 3 Department of Orthopaedic Surgery, Affiliated Hospital of Xiangnan University, Hunan 423000, China; 4 Department of Metabolism and Aging, The Scripps Research Institute, Jupiter, FL 33410, USA; 5 Department of Biochemistry, Molecular Biology and Biophysics, The Institute on the Biology of Aging and Metabolism, University of Minnesota, Minneapolis, MN 55455, USA; 6 Department of Orthopaedic Surgery, Brigham and Women's Hospital, Harvard Medical School, Boston, MA 02115, USA; 7 Department of Physical Medicine and Rehabilitation, University of Pittsburgh, Pittsburgh, PA 15213, USA; 8 Department of Neurology and Neurological Sciences, Stanford University School of Medicine, Stanford, CA 94305, USA; 9 Departments of Molecular Pharmacology and Medicine, Albert Einstein College of Medicine, Bronx, NY 10461, USA

**Keywords:** intervertebral disc, heterochronic parabiosis, systemic factors, proteoglycan, aging

## Abstract

Whether disc aging is influenced by factors beyond its local environment is an important unresolved question. Here we performed heterochronic parabiosis in mice to study the effects of circulating factors in young and old blood on age-associated intervertebral disc degeneration. Compared to young isochronic pairs (Y-Y), young mice paired with old mice (Y-O) showed significant increases in levels of disc MMP-13 and ADAMTS4, aggrecan fragmentation, and histologic tissue degeneration, but negligible changes in cellular senescence markers (p16^INK4a^, p21^Cip1^). Compared to old isochronic pairs (O-O), old mice paired with young mice (O-Y) exhibited a significant decrease in expression of cellular senescence markers (p16, p21, p53), but only marginal decreases in the levels of disc MMP-13 and ADAMTS4, aggrecan fragmentation, and histologic degeneration. Thus, exposing old mice to young blood circulation greatly suppressed disc cellular senescence, but only slightly decreased disc matrix imbalance and degeneration. Conversely, exposing young mice to old blood accelerated their disc matrix imbalance and tissue degeneration, with little effects on disc cellular senescence. Thus, non-cell autonomous effects of circulating factors on disc cellular senescence and matrix homeostasis are complex and suggest that disc matrix homeostasis is modulated by systemic factors and not solely through local disc cellular senescence.

## INTRODUCTION

Intervertebral disc degeneration (IDD) contributes to biomechanical dysfunction of the spine commonly associated with low back pain, resulting in tremendous socioeconomic burden [1, 2]. Aging is the leading risk factor for IDD [3, 4], which greatly contributes to chronic disability and debilitating pain in the elderly [5, 6]. With the rapid growth of the older adult population worldwide [7], understanding the biology of disc aging is imperative to develop effective treatment strategies for age-dependent IDD.

Aged discs exhibit many degenerative changes; most notably progressive loss of matrix proteoglycan (PG) leading to dehydration, tissue fibrosis, depressurization in the nucleus pulposus (NP), and decreased disc height [8]. Age-dependent loss of disc PG results from matrix homeostatic imbalance caused by increased matrix PG catabolism and decreased PG anabolism [8, 9]. Disc matrix imbalance is caused by the progressive loss of functional disc cells, likely stemming from increased apoptosis and cellular senescence during the course of aging. Growing evidence indicates that disc cellular senescence is a key factor responsible for disc PG loss and deterioration of the disc associated with aging. Cellular senescence is characterized by persistent genomic damage, growth arrest, and a senescence-associated secretory phenotype (SASP) comprised of pro-inflammatory cytokines and matrix metalloproteinases (MMPs) that can have profound impact on neighboring cells and the disc matrix [10, 11]. The causative role of cellular senescence in driving disc PG loss and disc aging was recently demonstrated whereby clearance of senescent cells ameliorated age-associated IDD using mice [12].

The predominant view of age-associated IDD is that it is caused by local, cellular changes within the disc [4, 13–15]. This view is based on the degenerative changes observed directly in aged discs [9, 16]. These observations include fissures and fibrosis at the tissue level, and oxidative damage, apoptosis, cellular senescence, and extracellular matrix (ECM) fragmentation at the molecular and cellular levels [9]. Time-dependent accumulation of stochastic damage to the resident disc cells stems from mechanical, nutritional, and inflammatory stress and is thought to underlie the disc cellular senescence. However, recent evidence suggests disc aging may be influenced by factors beyond the local disc environment. For example, osteopenia and osteoporosis correlates with increased incidences of IDD [17] and sclerosis reduces the nutrient transport to disc tissue [18]. In addition, muscular dystrophy has been shown to reduce PGs in the intervertebral disc (IVD), leading to acceleration of degenerative disc disease in a Duchenne muscular dystrophy model [19]. Although there is increasing evidence to suggest that aging is systemic, whether that is the case and to what extent cell non-autonomous factors may influence disc aging are important questions that have not been carefully investigated.

Here we tested the hypothesis that systemic blood-borne circulating factors play a role in regulating disc cellular senescence and age-associated IDD. We used a mouse model of heterochronic parabiosis, utilized previously to distinguish cell autonomous versus cell non-autonomous effects in aging cells tissues [20–22]. Using this approach, we observed improved PG homeostasis and reduced cellular senescence in the IVDs of old mice when exposed to a young environment. Conversely, perturbed disc PG homeostasis and increased disc cellular senescence were found in young mice exposed to old blood. These findings suggest that circulating factors significantly influence the disc aging phenotype as well as the rate at which age-associated IDD progresses. Identifying these factors and downstream effectors could provide novel target(s) for which to develop therapies to mitigate age-associated IDD.

## RESULTS

### Circulatory factors affect disc aggrecan gene expression

**Figure 1:**
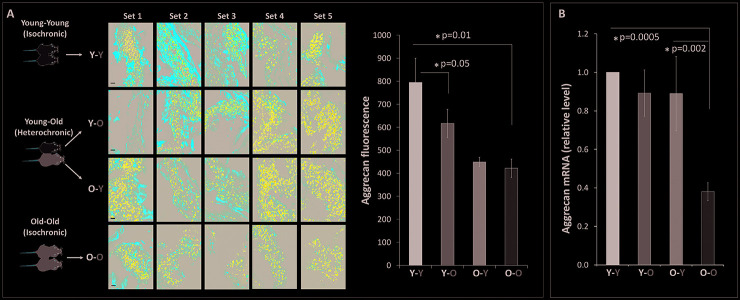
**Effects of circulatory factors on aggrecan mRNA and protein expression in intervertebral discs in mouse heterochronic parabionts.** (**A**) representative aggrecan immunofluorescent (red) images of nucleus pulposus (NP) tissue of the parabionts. Quantification of the NP aggrecan immunofluorescence is shown in the bar graph. (**B**) Disc aggrecan mRNA levels were quantified by qRT-PCR and normalized to GAPDH. Data shown are mean ± *SEM* of five independent experiments (n=5 per group), *p < 0.05. Scale bar = 20 μm.

Disc matrix PG loss is largely due to destruction of aggrecan, the major PG structural constituent of the disc ECM. The aggrecan core proteins function in maintaining the disc NP’s hydration and osmotic pressure. Aggrecan is an important biomarker of disc health as its loss directly correlates to age-associated IDD [8]. As expected, old mice (O-O) were found to have less disc aggrecan immunofluorescence than young mice (Y-Y) (Figure 1A). Compared to young mice in the isochronic pairing (Y-Y), young mice in the heterochronic pairing (**Y**-O) exhibited 21% decrease in disc aggrecan immunofluorescence (p<0.001). On the other hand, old mice in the heterochronic pairing (**O**-Y) were found to have slightly higher disc aggrecan immunofluorescence (0.82 ± 0.03) than the old mice (O-O) in the isochronic pairing (0.76 ± 0.04), but the increase was not statistically significant. These aggrecan immunofluorescence findings are consistent with disc aggrecan mRNA expression (Figure 1B), demonstrating that exposure to old circulatory factors suppressed aggrecan expression while exposure to young circulatory factors enhanced aggrecan expression in mouse IVDs.

### Circulatory factors affect disc aggrecanolysis and matrix metalloproteinase (MMP) gene expression

**Figure 2:**
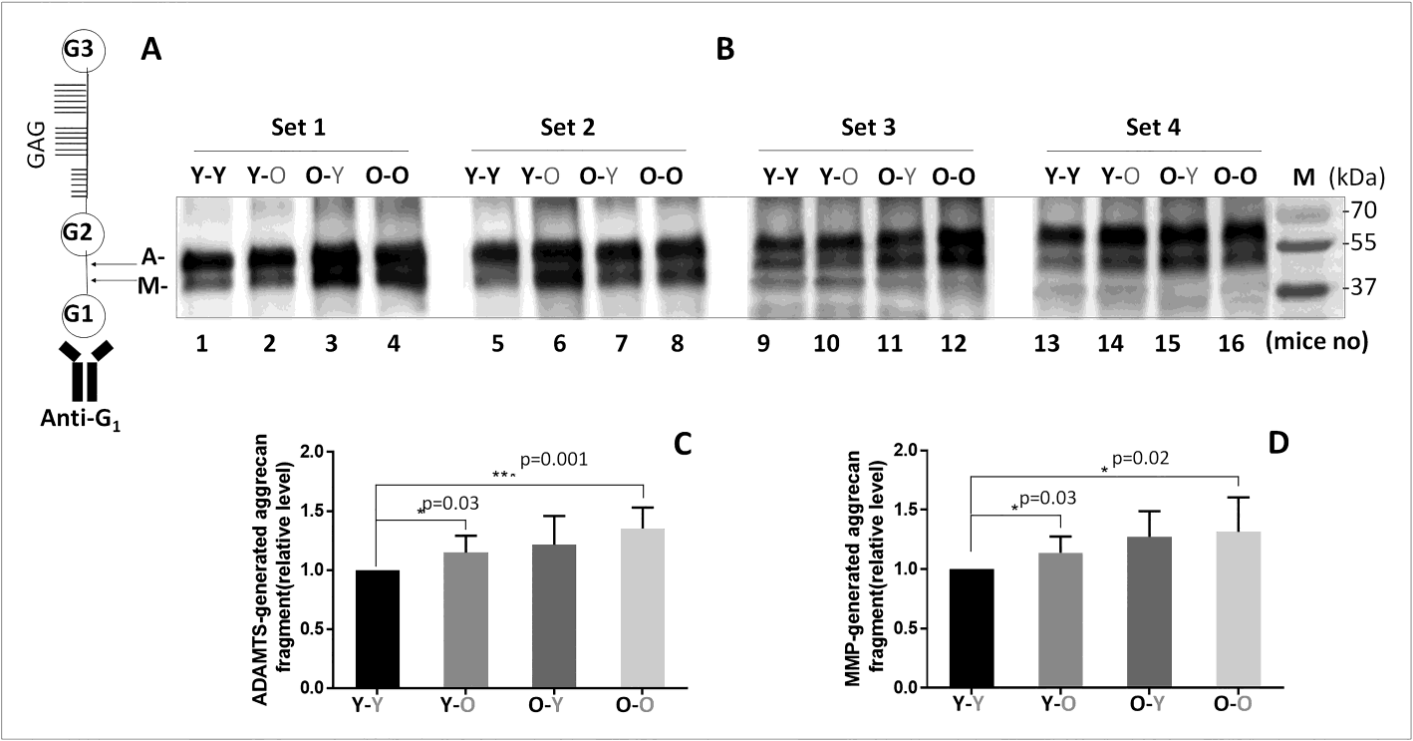
**Effects of circulatory factors on aggrecanolysis in IVDs from mouse heterochronic parabionts.** (**A**) A schematic of the mouse aggrecan core protein showing the three globular domains, G1, G2, G3, and sulfate-rich GAG region between G2 and G3. Anti-G1 was used to detect aggrecan and its fragments. The cleavage sites between G1 and G2 interglobular domains by ADAMTS (G1-NVTEGE392) and MMP (G1-VDIPEN360) proteases are indicated by the letter A and M, respectively. (**B**) Immunoblot of MMP- and ADAMTS-mediated cleavage of disc aggrecan of mice from the four groups. Graphs on right are quantification of aggrecan fragments shown in panel on left. Data shown are mean ± *SD* of 4 independent experiments, *p < 0.05.

**Figure 3:**
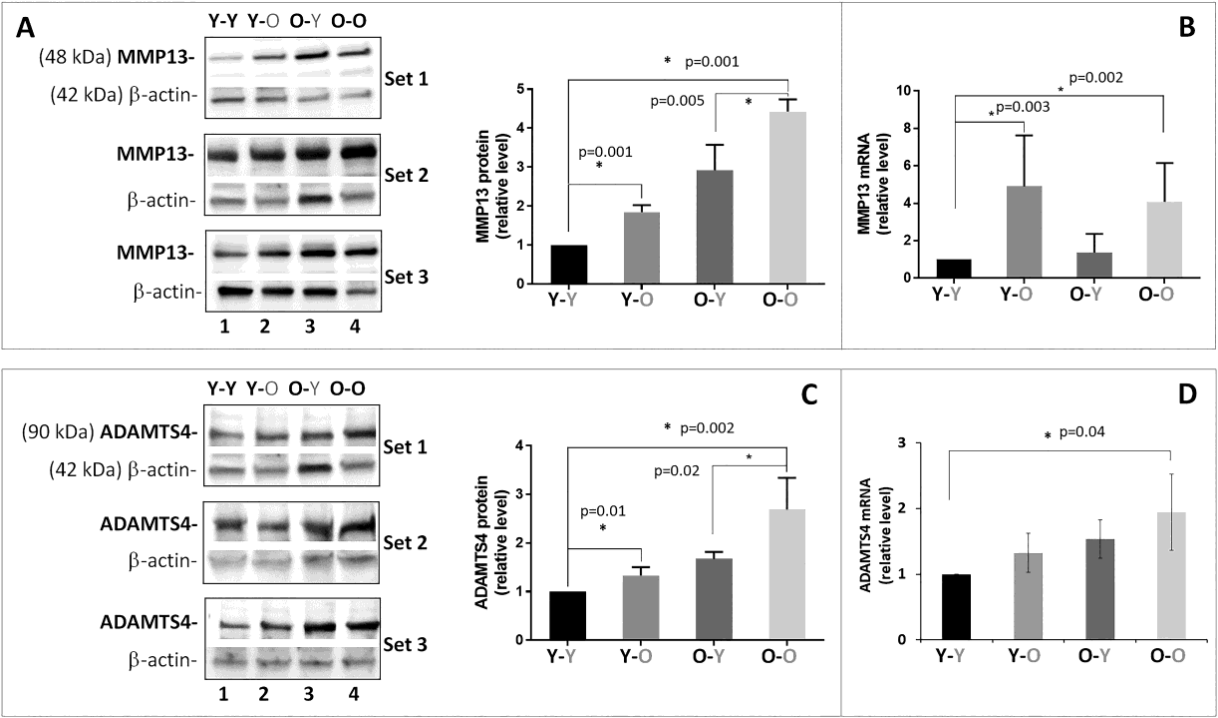
**Effects of circulatory factors on catabolic gene expression in intervertebral discs obtained from mouse heterochronic parabionts.** Levels of disc MMP13 protein (**A**) MMP13 mRNA, (**B**) ADAMTS4 protein, (**C**) ADAMTS4 mRNA, and (**D**) were quantified by Western blot and qRT-PCR. Graphs on the right of the Western blots show the relative levels of MMP13 and ADAMTS4 protein normalized to actin. Data shown are mean ± *SD* of 5 independent experiments for RT-PCR and 4 experiments for Western blot, *p < 0.05.

As expected, disc aggrecan fragmentation catalyzed by MMP and ADAMTS classes of MMPs was elevated in old mice (O-O) compared to young mice (Y-Y) (Figure 2). Likewise, young mice exposed to circulatory factors from old mice significantly increased both MMP- and ADAMTS-mediated disc aggrecanolysis (Y-Y versus **Y**-O). This finding is consistent with the enhanced mRNA and protein expression of MMP13 and ADAMTS4 in discs of **Y**-O mice compared to those in Y-Y mice (Figure 3). Disc MMP13 mRNA increased more than fourfold (Figure 3B) and its protein increased 85% (Figure 3A) in **Y**-O mice compared to Y-Y mice. Disc ADAMTS4 mRNA and protein also increased 20-30% (Figure 3C, 3D) in **Y**-O mice compared to Y-Y mice.

When old mice were exposed to young circulatory factors, disc aggrecanolysis descreased (**O**-Y versus O-O), but the differences between these two groups were not significant (Figure 2). This finding is also consistent with the reduced mRNA and protein expression of MMP13 (~30%, Figure 3A, 3B) and ADAMTS4 (15-30%, Figure 3C, 3D) in discs of **O**-Y mice compared to those in O-O mice. While these effects were seen in the heterochronic pairing for eight weeks, it should be noted that reduced aggrecan fragmentation was also seen in 4-week parabiosis (Supplementary Figure 2), but the effects were less pronounced, suggesting that prolonged exposure to young circulatory factors is needed to suppress aggrecanolysis in the aged disc.

### Circulatory factors affect disc cellular senescence and SASP

**Figure 4:**
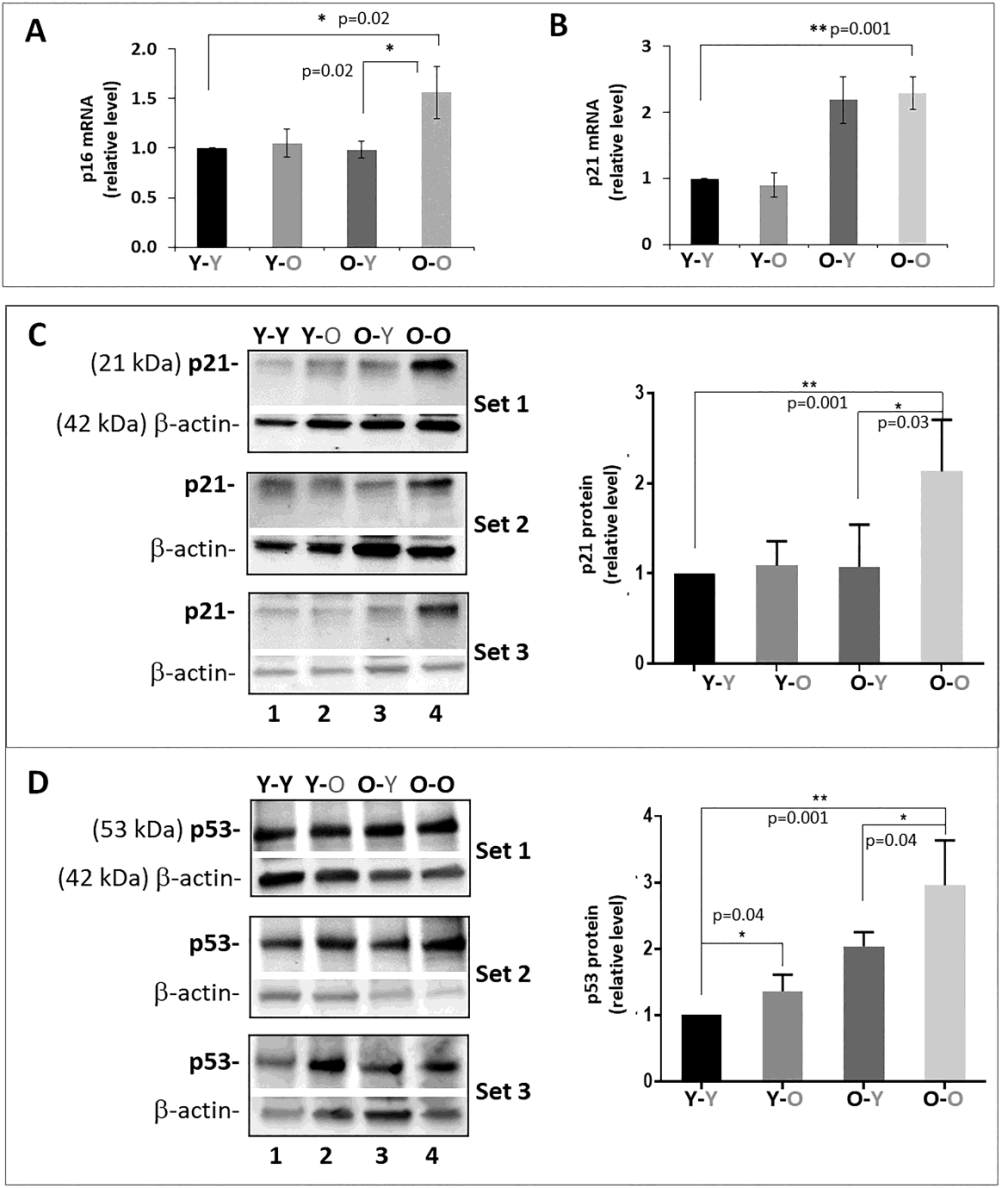
**Effects of circulatory factors on expression of cellular senescence markers in IVDs in mouse heterochronic parabiotants.** qRT-PCR quantitation of disc *p16^INK4a^* (**A**) and *p21^Cip1^* (**B**) mRNAs. Western blot of p21^Cip1^ (**C**) and p53 (**D**) protein. Graphs on the right of the Western blot showed quantitation of these proteins normalized to actin. Data shown are mean ± *SD* of 5 independent experiments for RT-PCR and 4 experiments for Western blot, *p < 0.05. **p < 0.01.

Enhanced aggrecanolysis and catabolic gene expression observed in **Y**-O mice compared to Y-Y mice could be due to increased disc cellular senescence. This is because senescent disc cells exhibited perturbed matrix homeostasis, which serves as compelling evidence of the paracrine and endocrine effects of senescence-associated secretory phenotype (SASP) on inducing senescence and matrix catabolism in distal tissues. To test this, we assessed the level of disc cellular senescence by measuring three key markers of cellular senescence: p16^INK4a^, p21^Cip1^, and p53 [12]. As expected, expression of all three markers was elevated in the discs of old mice (O-O) mice, compared to young mice (Y-Y) (Figure 4). When exposed to old blood, the discs of heterochronically-paired young mice (**Y**-O) displayed a significant increase in p53 protein expression compared to isochronically paired young mice (Y-Y). Discs of heterochronically-paired young mice (**Y**-O) also trended toward an increase in p21 and p16^INK4a^ proteins compared to those in isochronically-paired young mice (Y-Y), although the changes were not statistically significant.

**Figure 5:**
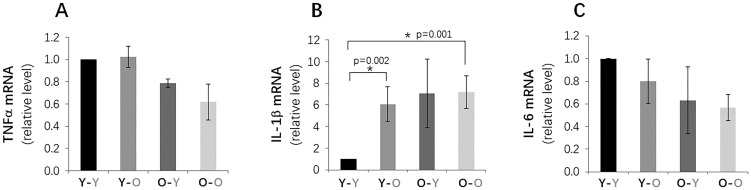
**Effects of circulatory factors on gene expression of key SASP factors in IVDs in mouse heterochronic parabiotants.** qRT-PCR quantitation of TFNα (**A**), L-1β (**B**) and IL-6 (**C**) mRNAs from discs from the four groups of mice. Data shown are mean ± *SD* of 5 independent experiments, *p < 0.05.

To further evaluate other senescence biomarkers, we measured and compared the mRNA expression levels of several key SASP factors in disc tissue, including TNFα, IL-1β, and IL6. Disc IL-1ß gene expression was significantly greater in isochronically-paired old mice (O-O), compared to isochronically-paired young mice (Y-Y), as expected (Figure 5). Unexpectedly, TNFα and IL-6 mRNA levels were slightly lower in old mice (O-O), compared to young mice (Y-Y). Exposure to old blood also unexpectedly decreased IL-1ß gene expression in disc tissue of heterochronically-paired young mice (**Y**-O), compared to Y-Y mice. No significant changes in disc TNFα or IL-6 expression were observed between the **Y**-O and Y-Y mice. These findings suggest that exposure to an old environment only had modest influences on disc cellular senescence, and the differential effects of old blood on expression of different SASP factors in young mice highlight the complex interaction between the disc tissue and its surrounding systemic environment.

Levels of *p16^INK4a^* mRNA and p21^Cip1^ and p53 proteins in the IVDs of old mice exposed to young blood (**O**-Y) were all lower than those measured in isochronically-paired old mice (O-O) (Figure 4). Based on these measurements, it was expected that expression of SASP factors would also be reduced in disc tissue of **O**-Y compared to O-O mice. However, only disc IL-1β mRNA was found to be lower in **O**-Y than O-O mice, whereas no significant changes were observed for TNFα and IL6 gene expression. These results suggest that exposure to young circulatory factors suppressed markers of senescence burden in disc tissue of aging mice, but not necessarily expression of the pro-inflammatory cytokines.

### Circulatory factors affect disc histologic features

**Figure 6:**
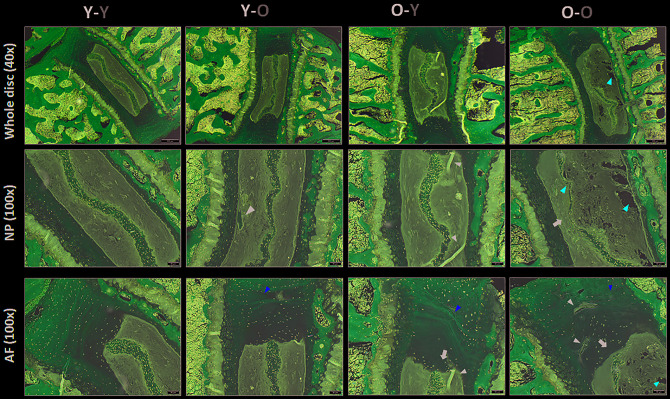
**Impact of circulatory factors on gross disc morphology.** H&E staining of lumbar disc was performed to assess the gross morphological changes with aging. Compared to discs of Y-Y mice, discs of O-O mice exhibited increasing loss of a distinct NP/AF boundary (black arrows), loss of AF structure in which the AF lamellae become less concentric and more serpentine, with each lamellae spaced farther apart (yellow arrowheads), loss of NP matrix, indicated by large empty space gaps (red arrowheads) and fissures/clefts (black arrowheads). These degradative changes were also observed in Y-O mice but blunted in the O-Y mice, suggesting the global influences of blood factors on disc aging phenotype. Disc sections from four representative mice of each group (Y-Y, Y-O, O-Y, O-O) are shown. Scale bar = 50μm of H&E stained disc sections.

To determine whether blood circulatory factors affect disc degenerative changes in mice at the tissue level, we performed H&E histology on lumbar discs (L4-L5) to compare the gross morphological disc features among groups. We quantified the changes in several key disc histological features using a grading system previously established by Tam et al. for mouse discs [23] in which higher histologic scores correspond to greater degenerative changes. Histology grading was done in a blinded manner by three scorers on multiple H&E-stained disc sections. Compared to isochronically-paired young mice (Y-Y), isochronically-paired old mice (O-O) exhibited degenerative histological changes in their discs. These included increased AF clefts/fissures, loss of NP cellularity, loss of a well-defined AF/NP boundary, thinning and loss of NP matrix, and structural disorganization of AF lamellae (Figure 6). Most of these degenerative changes, with the exception of the AF/NP boundary parameter, were elevated in discs of young heterochronic parabionts (**Y**-O), compared to the young isochronic pairs (Y-Y) (Figure 6 and Table 1), suggesting that exposure to old blood accelerates the disc aging phenotype.

**Table 1:** Intervertebral disc histological scores.

**Score (mean ± SEM)**	**Y-Y**	**Y-O**	**O-Y**	**O-O**
NP cellularity loss	0.2±0.6	1.1±0.7	0.8±0.8	2.2±0.6
NP clefts/fissures	1.0±0.6	1.3±0.7	1.1±0.6	1.5±0.8
AF/NP boundary	0.7±0.8	0.5±0.5	0.8±0.6	0.7±0.5
AF disorganization	0.4±0.8	1.2±0.7	1.6±0.7	1.4±0.8
AF clefts/fissures	0.0±0.0	0.6±0.7	0.8±0.8	1.2±0.6
Composite score	2.3±1.4	4.7±1.5	5.1±1.6	7.0±1.5

Some histological degenerative changes, particularly NP cellularity loss and fissures, were reduced in the disc tissue of old mice exposed to young blood (**O**-Y), as compared to isochronically-paired old mice (O-O) (Figure 2 and Table 1). However, while disc histological features tended to improve in old mice paired with young mice, the individual and composite scores did not differ significantly between O-O and **O**-Y mice. Together, these results suggest that the disc aging phenotype can be influenced by systemic factors.

### Circulatory factors affect disc tissue aging in accelerated aging Ercc1^-/Δ^ mice

**Figure 7:**
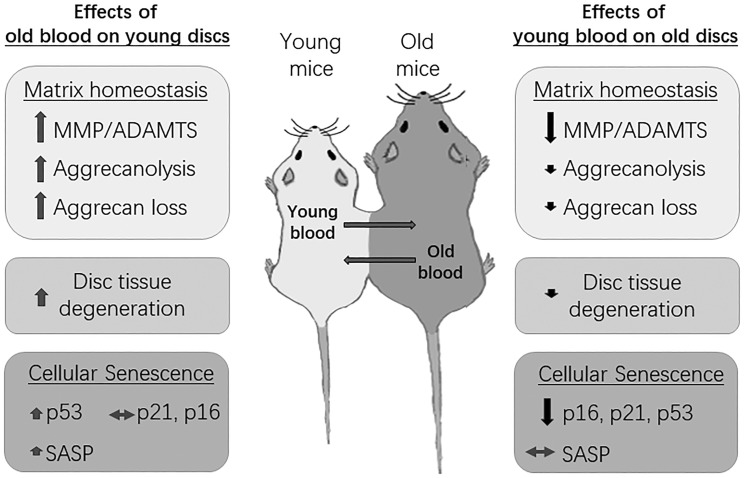
**Summary of the effects of circulatory factors on the disc aging phenotype.** Young mice exposed to old blood exhibited a small, albeit insignificant increase in disc cellular senescence, but a significant increase in disc matrix PG imbalance and tissue degeneration. Old mice exposed to young blood showed a significant decrease in disc cellular senescence, but only a modest improvement in disc matrix PG homeostasis and tissue morphology. Black arrows=decreasing effects; red arrows=increasing effects; grey arrows=no effects. Magnitude of effect is indicated by size of arrow.

DNA repair-deficient *Ercc1^-/Δ^* mice age rapidly and exhibit many age-related disorders, including accelerated disc aging due to persistent DNA damage. Past studies have reported on the health rejuvenation and extended lifespan of these mice upon systemic injection of muscle derived stem cells (MDSCs) [24]. Because exposure to young blood mitigates age-related IDD in natural aging mice, we investigated if similar effects occurred in *Ercc1^-Δ/^* mice with accelerated aging. Indeed, compared to isochronically-paired old *Ercc1^-/Δ^* mice, *Ercc1^-/Δ^* mice from a heterochronic pairing with young Wt mice for four weeks also exhibited improved histological features (Supplementary Figure 1A), greater aggrecan immunofluorescence in their disc NP (Supplementary Figure 1A, 1B) and higher total NP GAG content (Supplementary Figure 1C). Loss of laminB1 expression is a marker of cellular senescence [25]. Discs of *Ercc1^-/Δ^* mice from the heterochronic pairing also contained higher levels of laminB1 in their AF and NP (Supplementary Figure 1D), suggesting reduced disc cellular senescence in these mice after being exposed to young blood. The results indicate that exposure to young systemic factors blunt age-associated IDD in natural as well as accelerating aging mice. The effects of systemic circulatory factors on a disc aging phenotype in mice are summarized in Figure 7.

## DISCUSSION

It has become increasingly appreciated that cell non-autonomous factors can contribute to tissue and cellular aging at many sites. However, it is unclear how age-dependent IDD is influenced by an old systemic environment. Moreover, the relative contributions of systemic and local factors to disc aging are not known. Earlier experiments involving whole body exposure to genotoxic stress—e.g. global knockdown of a DNA repair enzyme *ERCC1*, or whole-body exposure to genotoxic stress, such as ionizing radiation—accelerated age-related IDD in mice [26, 27]. Because these interventions simultaneously impact the local disc tissue as well as tissues at other sites, it is not possible to differentiate the relative contributions of local niche effects versus global influences on disc aging in these studies. Nevertheless, they can inform as to whether factors acting extrinsic to the disc per se contribute to the aging phenotype. Here, using heterochronic parabiosis in mice, we demonstrate that age-associated IDD is indeed greatly impacted by systemic circulatory factors. In particular, old blood promotes disc degenerative changes in young mice while young blood only modestly improves disc health in old mice. Our findings are consistent with past studies implicating the contribution of global effects on disc aging and degeneration, including the positive correlations between IDD and several non-disc tissue disorders such as osteoporosis, Duchenne muscular dystrophy, and atherosclerosis [17–19].

### Effects of old circulatory factors on young discs

Similar to previous results, the IVDs in old mice exhibited tissue level degeneration and perturbed proteoglycan matrix homeostasis, including elevated aggrecanolysis and aggrecan loss as typically seen in humans and other animals [8]. These degenerative changes, while absent in young mice, appeared in young mice sharing blood circulation with old mice for just ~eight weeks in our heterochronic parabiosis model. For instance, disc aggrecan immunofluorescence decreased while aggrecanolysis increased in **Y**-O mice compared to Y-Y mice. In addition, greater histological degenerative features, especially loss of NP cellularity and AF lamella disorganization, were found in the **Y**-O mice than Y-Y mice. It should be noted, however, that these disc degenerative features were less pronounced in four-week (Supplementary Figure 2) heterochronic parabiosis compared to pairing for eight weeks. Thus, prolonged exposure to old blood factors accelerate disc matrix PG imbalance and tissue degeneration in young mice. These findings are consistent with the previous blood infusion and heterochronic parabiosis studies that reported inhibitory and detrimental impacts of old blood on multiple tissues, including muscle, liver, and brain hippocampus [28].

Cellular senescence has recently emerged as a major driver of disc aging. Senescent disc cells have been demonstrated to acquire a phenotype that disrupts PG homeostasis [13, 29, 30]. Systemic clearance of cellular senescence also mitigates age-associated IDD in mice [12]. Thus, one possible mechanism of action of old blood on a young disc is the induction of disc cellular senescence. However, exposure to old blood neither significantly increased expression of the key markers of cellular senescence, e.g. p16^INK4a^ or p21^Cip1^, nor induced expression of the key SASP factors such as IL-1β, IL-6, or TNFα. Hence, the negative effects of old blood on young disc must be mediated through mechanisms other than disc cells becoming senescent. This contrasts with the effects of heterochronic parabiosis on senescence observed in liver, kidney, and lung tissues where significant up or down regulation of senescence and SASP markers were significantly affected by circulating factors [31]. Part of this difference could be due to clearance of senescent cells by young immune cells in tissues other than the disc and/or to the lower concentrations of the key circulating SASP factors, which drive paracrine senescence, within the poorly vascularized disc. However, the discs of **Y**-O mice did have higher levels of ADAMTS4 and MMP13 proteins than the discs of Y-Y mice, but whether the sources of these proteins are exogenous or endogenous requires further study. While it is possible that these catabolic proteins were derived from old blood and entered the disc tissue from the systemic environment, increased mRNAs of ADAMTS4 and MMP13 in **Y**-O discs suggest that at least part of the increase was due to production by the resident disc cells. The specific humoral factors in old blood responsible for upregulating gene expression of disc ADAMTS4 and MMP13, as well as circulating factors responsible for mediating aggrecanolysis and promoting aggrecan loss in young disc remains to be resolved.

While the implications of this study suggest that circulating factors may exist that can be either restored or inhibited in old blood decelerate IDD and/or restore disc integrity, the identity of such factor(s) remain to be identified. Previous heterochronic parabiosis studies reported specific circulatory soluble factors that closely correlate with the effects on specific tissue. For example, the chemokine CCL11 (eotaxin) and ß2M were identified in old blood as able to impair neurogenesis in the brains of young heterochronic parabiont mice [32]. On the other hand, loss of normal cardiac function in an aging heart, leading to cardiac hypertrophy and diastolic heart failure, was reported to be partially due to the lack of certain circulating factors in old mice. One of these factors was identified as the growth differentiation factor 11 (GDF11), which was initially reported to be reduced in the old circulation, though this remains complex and controversial [20]. Clearly, the identification of the specific humoral factors or cells in the circulation that regulate the disc environmental niche, its resident cells, and matrix homeostasis will be important.

### Effects of young circulatory factors on old mouse discs

Old mice exposed to young blood for a short (4 weeks, Supplementary Figure 2) or longer (8 weeks, Figures 1–3) duration in our heterochronic parabiosis showed minimal evidence of rejuvenation in their disc proteoglycan matrix health. Specifically, there was negligible improvement in disc aggrecan immunofluorescence and an insignificant decrease in MMP- and ADAMTS-mediated disc aggrecanolysis in **O**-Y mice, compared to O-O mice. Similarly, exposure to young blood did not significantly improve disc histologic features in the old heterochronic parabionts, apart from the loss of NP cellularity (Figure 6 and Table 1). It is interesting to note that while exposure to young blood significantly blunted the loss of NP cellularity in old mice, such effect did not translate to overall disc aggrecan content (Figure 1), possibly because NP cells in these old mice do not actively synthesize PG matrix or the rate of production was too slow to produce a measurable difference after eight weeks of parabiosis. Because blood circulation is not fully shared until 2-3 weeks after surgical pairing, a longer parabiosis duration, e.g. 12-16 weeks, might be necessary to increase beneficial effects of young blood on old discs.

The lack of rejuvenation of old discs by young blood could also be due to irreversible changes conferred by aggrecanolysis and the substantial loss of disc aggrecan in 18-month-old mice prior to being paired with the young mice. Aggrecanolysis, the proteolytic fragmentation of aggrecan, is irreversible and leads to the eventual loss of aggrecan from disc tissue [33]. Hence, performing the heterochronic parabiosis on younger mice (<18 months old) and for a longer duration might be necessary to prevent or delay this age-related degenerative change. Nevertheless, rejuvenation of cognitive function and synaptic plasticity by young blood has been reported in mice when they were heterochronically-paired at 18 months of age [34]. Additionally, young blood rejuvenates hepatic progenitor cells and skeletal muscle stem cells even in older mice (19-26 months) when they were first paired with young mice [35]. Circulating peripheral blood cells contributed to meniscus repair in wildtype rats sharing blood with transgenic rats [22]. Together with our findings, the beneficial impacts of heterochronic parabiosis are marginal for IVDs and neurogenesis, while more notable effects have been noted for liver, cardiac hypertrophy, fracture, and skeletal muscle repair—robust for muscle and modest for liver and meniscus. These findings underscore the complex and nonuniform nature of interactions between different organs and their systemic environment, as well as the nature of the studies, e.g. parabiosis duration, genetic strains used, tests performed, etc.

While young blood only had marginal effects on restoring old disc matrix homeostasis, it significantly reduced disc cellular senescence in the old mice. Levels of all three key protein markers of cellular senescence (p16, p21, and p53) were decreased in disc tissue of O-Y mice compared to O-O mice, a result similar to what we have observed in liver, kidney, and lung of the paired mice [31]. In addition, MMP13, and ADAMTS4 were also greatly reduced in **O**-Y compared to O-O mice. How young blood decreases these proteins in the IVDs of old mice is not understood, although dilution of old blood factors by young blood could be a mechanism if these SASP factors enter the disc from the systemic environment. Otherwise, if MMP13, and ADAMTS4 are produced by the resident disc cells, then their expressions in the niche of old disc tissue must be down regulated by some circulating factors from the young mice.

### Effects of young blood on discs of accelerated aging mice

Interestingly, while exposure to young blood only marginally mitigated age-related IDD in natural aging mice, it significantly reduced disc degeneration in accelerated aging *Ercc1^-/Δ^* mice. *Ercc1^-/Δ^*mice paired with young mice for only four weeks had about twice the level of disc NP aggrecan immunofluorescence and 40% greater NP GAG content compared to the isochronic paired *Ercc1^-/Δ^* mice. The positive effects could be due to accelerated aging of *Ercc1^-/Δ^* mice, which have a compressed average lifespan of 5-6 months, or about four times shorter than naturally aging mice [36]. Thus, a 4-week parabiosis of *Ercc1^-/Δ^* mice would be equivalent to a 16-week parabiosis of natural aging mice, a sufficient duration for young blood to confer health benefit to disc tissue in these animals. The beneficial effects of young blood on *Ercc1^-/Δ^* mice in our heterochronic parabiosis are consistent with previous findings demonstrating extension of lifespan and healthspan in these progeroid mice through multiple systemic interventions [24, 37–39]. These interventions include inhibition of IKK/NF-kB signaling, treatment with senolytics such as an HSP90 inhibitor or the combination of dastinbi and quercetin, and treatment with adult stem cells from young, but not old mice.

### Unusual and unexpected findings

Our heterochronic parabiosis produced several unexpected results. First, there was no direct correlation between disc aggrecanolysis and the expression levels of two key matrix metalloproteinases (MMPs)—ADAMTS4 and MMP-13. Exposure of young mice to old blood greatly increased both disc aggrecanolysis and expression of ADAMTS4 and MMP-13, suggesting that these MMPs are responsible for disc aggrecanolysis. In contrast, exposure of old mice to young blood marginally reduced disc aggrecanolysis, but significantly decreased disc expression of ADAMTS4 and MMP-13. This suggests that either MMPs other than these are responsible for disc aggrecanolysis or substantial aggrecanolysis already happened in the old mice before pairing. Second, there is a disconnect between the disc aggrecan mRNA level and aggrecan protein level in **O**-Y mice compared to O-O mice. While disc aggrecan mRNA was greatly upregulated, its protein level (aggrecan immunofluorescence) was only slightly increased when old mice were paired with young mice (Figure 1). This observation suggests that young blood exposure primarily upregulates transcription of disc aggrecan but not the post-transcription steps such as translation, post-translation modifications, export to the ECM, etc. Third, expression of SASP factors is highly variable in disc tissue of old mice. For example, only IL-1β, not IL-6 and TNFα, were found to be expressed significantly more in the disc of 20-month old mice (O-O) compared to young mice (Y-Y). This is unexpected as all these three cytokines were found in elevated levels in degenerative and aged human discs [40–42]. Additionally, exposure of old mice to young blood did not affect their disc IL-1β gene expression while exposing young mice to old blood significantly increased their disc IL-1β gene expression. Altogether, these unexpected findings suggest that the effects of systemic circulatory factors on disc matrix homeostasis and cellular activities are highly complex.

It should be noted that biomechanical considerations cannot be completely ruled out as contributors to our results, and that this could be addressed in part via Sham controls in which heterochronic pairing are surgically done without shared blood circulation. However, this is unlikely to be a major confounder in our data for a few key reasons. First, our 4 and 18 month old mice were chosen strategically as this is an age in which the long bones are developed in young mice making the union more successful, whereas the older mice are not yet too advanced in age. Second, the body size of younger (~30-35g) and older (~40-45g) mice only vary slightly, based mostly on differences in adiposity. By the time animals were necropsied at 6 and 20 mo of age, respectively, these differences were even smaller. Second, we also did not typically observe any noticeable impact or dominance between parabionts on ambulation, as animals typically do not “drag” each other around, but instead become increasingly synchronized in terms of sleeping, eating and moving in unison within days of surgery. For these reasons, the notion of “young animals carrying around the old mice”, an observation we did not really observe in this model, as driving the disc phenotypes seemed unlikely.

## CONCLUSIONS

Leveraging heterochronic parabiosis models, we demonstrated that exposure to old blood greatly accelerates disc matrix degeneration in young mice. Conversely, we also found that a young circulatory blood environment only modestly slows the progression of disc matrix degeneration in old mice; we speculate that longer duration of parabiosis might be needed to confer greater benefits or that parabiosis needs to be performed on less aged mice to delay or prevent disc aging more effectively. These findings support the hypothesis that disc aging is influenced by global changes and not just by the local cell autonomous changes in the disc. These findings also implicate that effective treatment of IDD in older individuals requires consideration of the contributions of systemic aging factors on disc health. Our results also suggest the existence of one or more blood-borne systemic factors or cells controlling the disc aging process, once identified may represent therapeutic targets to mitigate age-associated IDD.

Future efforts are now needed to elucidate the geronic factor(s) that drive the disc aging process, which once identified, may represent promising therapeutic targets to mitigate age-associated IDD.

## MATERIALS AND METHODS

### Parabiosis of Wt mice

Young (Y, aged 4 months) and old (O, aged 18 months) wildtype C57BL/6 male mice were obtained from the NIA-aged rodent colony for parabiosis and three mouse pairs were generated: young-young (Y-Y), young-old (Y-O), and old-old (O-O). Parabiosis surgery was performed as described in the Einstein Health Span Core [43, 44]. In brief, an incision was made from lateral aspect of the elbow to the knee to separate the skin from the fascia layer, this allowed for fusion of the skin as well as part of the peritoneum, in order to increase the area for blood exchange. Mice were kept one pair per cage following surgery and provided with adequate food and water. Mice were sacrificed approximately eight weeks after surgical pairing by exsanguination under isoflurane anesthesia. Spines and tails were isolated from the pairings and separated into four groups (Young Isochronic (**Y**-Y), Young Heterochronic (**Y**-O), Old Isochronic (**O**-O), and Old Heterochronic (**O**-Y), and were immediately frozen using liquid nitrogen and stored at -80°C until study. Of note, **O**-Y refers to the old mouse from the heterochronic pair while **Y**-O refers to the young mouse from the heterochronic pair. Parabiosis and related procedures were approved by the Einstein Institutional Animal Care and Use Committee (IACUC).

### Parabiosis of Ercc1^-/Δ^ mice

*Ercc1^-/Δ^* mice exhibit aging symptoms by the age of 5 weeks. Therefore, older 8-week-old female *Ercc1^-/Δ^* mice were surgically paired with younger 6-week-old female f1 Wt mice (C57BL/6:FVB/n) for 4 weeks before being sacrificed for spine isolation and analyses.

### Histological stain

Isolated lumbar spines segments were decalcified and embedded in paraffin (*Tissue Tek* processor and *Leica* embedder, ID:17492, Rankin, Holly, MI). Seven-micrometer sections were stained with hematoxylin and eosin (H&E) by standard procedures [26], then photographed under 40-100x magnification (*Nikon Eclipse* Ts100, Tokyo, Japan). Changes in histological features were evaluated by three blinded scorers using the histological grading system established for mouse discs [23] from the four groups. Three lumbar sagittal sections from each mouse were scored for the following specific features in the NP and anulus fibrosus (AF) as specified by Tam et al [23]. Normal young disc tissue is defined as having: (1) high NP cellularity that is centralized as a single mass of vacuolated cells enclosed within a layer of matrix; (2) AF that is aligned in concentric lamellae without disruption to the structure; (3) an NP matrix structure in which the matrix mass is continuous and full without significant empty space; and (4) a well-defined AF/NP boundary whereby the AF and NP compartments are always distinct. Each feature was scored individually with a score of 0–14; whereby, 0 represented the young health state and 14 represented the maximum degenerative state. A composite score was also calculated by adding all the individual scores (Table 1).

### Immunofluorescence

Mouse lumbar IVDs were isolated and fixed for two hours at 4°C in 2% paraformaldehyde. The tissues were cryoprotected with 30% sucrose in phosphate buffered saline (PBS, Cat.No.08118006, ThermoFisher Scientific, Pittsburgh, PA) overnight at 4°C, and then embedded in OCT (Cat.No. 4583, *Tissue-Tek,*Torrance, CA). Serial axial plane lumbar disc cryosections were cut at a thickness of 7 μm. The tissue sections were rehydrated in PBS, permeabilized, and blocked with 0.25% Triton X-100, 10% goat serum and 1% Bovine Serum Albumin (BSA, Cat.No.166099, ThermoFisher Scientific, Pittsburgh, PA) in PBS for 30 minutes at room temperature. Incubation with the specific primary antibodies (anti-Aggrecan, Cat.No. AB1031, Millipore; anti-MMP13, Cat.No. ab39012, Abcam, Cambridge, MA; anti-ADAMTS4, Cat. No. 185722, abcam, Cambridge, MA) were carried out overnight at 4°C following blocking. The sections were then incubated in a secondary antibody Cy3-conjugate goat anti-rabbit IgG (Cat.No.130233, Jackson Laboratory, West Grove, PA) solution for one hour according to the manufacturer’s protocol. Immunostained sections were imaged and analyzed under the Nikon A1 confocal laser microscope and NIS-elements microscopy imaging software.

### 1,9-Dimethylmethylene BLUE (DMB) colorimetric assay for sulfated Glycosaminoglycans (GAGs)

NP tissue was isolated and pooled from six lumbar IVDs of each mouse. The pooled tissue sample was digested using papain at 60°C for two hours. GAG content was measured in duplicate by DMB procedure using chondroitin-6-sulfate (Sigma, Milwaukee, WI, C-8529) as a standard. The DNA concentration of each sample was measured using the PicoGreen assay (Molecular Probes, Sunnyvale, CA) and used to normalize the GAG values. Average values from six reaction samples (two duplicates × three mice per group) were calculated +1 SE.

### Western blot analysis

The cervical and thoracic discs from each mouse were used to assess aggrecan fragmentation utilizing a previously established method [45] using anti-aggrecan primary antibody (Cat.No. ab36861, Abcam, Cambridge, MA) and anti-rabbit-HRP secondary antibody (Cat.No. 31460, ThermoFisher Scientific, Pittsburgh, PA). Protein from the spine disc samples was extracted using 4M guanidinium chloride as reported previously [45]. All samples were measured for total protein concentration using a BCA Protein Assay Kit (ThermoFisher Scientific, Pittsburgh, PA) to ensure equal loading. Loading buffer was added to 30 μg protein and separated on 4-20% SDS-PAGE gels before transferred onto PVDF membranes (Bio-Rad, Hercules, CA). The blots were blocked using 5% non-fat dry milk (Bio-Rad, Hercules, CA) at room temperature, shaken for one hour, and then incubated overnight with primary antibodies using MMP13 (Cat.No.39012, Abcam, Cambridge, MA), ADAMTS4 (Cat.No.185722, Abcam, Cambridge, MA), p16 (Cat.No.189034, Abcam, Cambridge, MA), p21 (Cat.No. 109199, Abcam, Cambridge, MA), p53 (Cat.No.1C12, ThermoFisher Scientific, Pittsburgh, PA), and β-actin (Cat.No. PA1-183, ThermoFisher Scientific, Pittsburgh, PA). The following day, the blots were washed three times with PBS-T buffer then incubated with the anti-rabbit-HRP secondary antibody (Cat.No. 31460, 1:10000, ThermoFisher Scientific, Pittsburgh, PA) for one hour at room temperature. Following another three washes with PBS-T, the blots were viewed with the *LiCoR Odyssey* imager (LI-COR Biosciences, Lincoln, NE) to visualize and quantifiy the protein bands.

### mRNA analysis

Total RNA from whole IVD tissue of each specimen’s lumbar vertebrae (L1-3) was isolated using TRIzol™ Reagent per manufacturer’s instructions (Cat. No. 15596026, ThermoFisher Scientific, Pittsburgh, PA). Real-time quantitative RT-PCR was run using iTaq™ Universal SYBR® Green One-Step Kit (Cat. No. 1725151, BIO-RAD, Hercules, CA) and Bio-Rad iCyclyer IQ5 detection system. Target gene expression was calculated by the comparative C_T_ method (ΔΔ C_T_) and normalized to GAPDH mRNA expression. The housekeeping gene GAPDH was chosen because its mRNA expression in disc tissue remains unchanged in aging and degenerating disc [46]. PCR primers used in the study are provided in Table 2.

**Table 2:** Primers used for RT-PCR analysis of gene expression.

**Gene**	**Forward (5’—3’)**	**Reverse (5’—3’)**
*GAPDH*	ACCCACTCCTCCACCTTTGAC	TCCACCACCCTGTTGCTGTAG
*Aggrecan*	AAGAATCAAGTGGAGCCGTGTGTC	TGAGACCTTGTCCTGATAGGCACT
*p16^INK4a^*	AATCTCCGCGAGGAAAGC	GTCTGCAGCGGACTCCAT
*p21*	GGAGACTCTCAGGGTCGAAA	GGATTAGGGCTTCCTCTTGG
*MMP13*	TCCCTGCCCCTTCCCTATGGT	CTCGGAGCCTGTCAACTGTGGA
*ADAMTS4*	ATGGCTATGGGCACTGTCTC	GTGTTTGGTCTGGCACATGG
*IL-1β*	GCAACTGTTCCTGAACTCAACT	ATCTTTTGGGGTCCGTCAACT
*IL-6*	GACTTCCATCCAGTTGCCTTC	ATTTCCACGATTTCCCAGAG
*TNFα*	AACAGAAACTCCAGAACATCTTGG	GTCGCGGATCATGCTTTCTG

### Statistical analysis

Shapiro-Wilk test was used to test for normality. Student's independent t test was used to analyze data that were found to be normal. For two-variable nonparametric data, the Mann-Whitney test was used. Analysis of variance (ANOVA) with Bonferroni correction for multiple comparison was used in cases of data with multiple experimental groups. Statistics were derived using GraphPad Prism from GraphPad Software (San Diego, CA). All graphs show mean values with error bars. Unless specified otherwise, p< 0.05 was considered significant.

## Supplementary Material

Supplementary Figures
