# Supplementary material for: Influences of circulatory factors on intervertebral disc aging phenotype
Source: Aging (Albany NY). 2020 Jun 11;12(12):12285–304. doi: 10.18632/aging.103421 (PMC7343497; doi:10.18632/aging.103421)
Supplement: Supplementary Figures [file aging-12-103421-s001..pdf]

SUPPLEMENTARY FIGURES

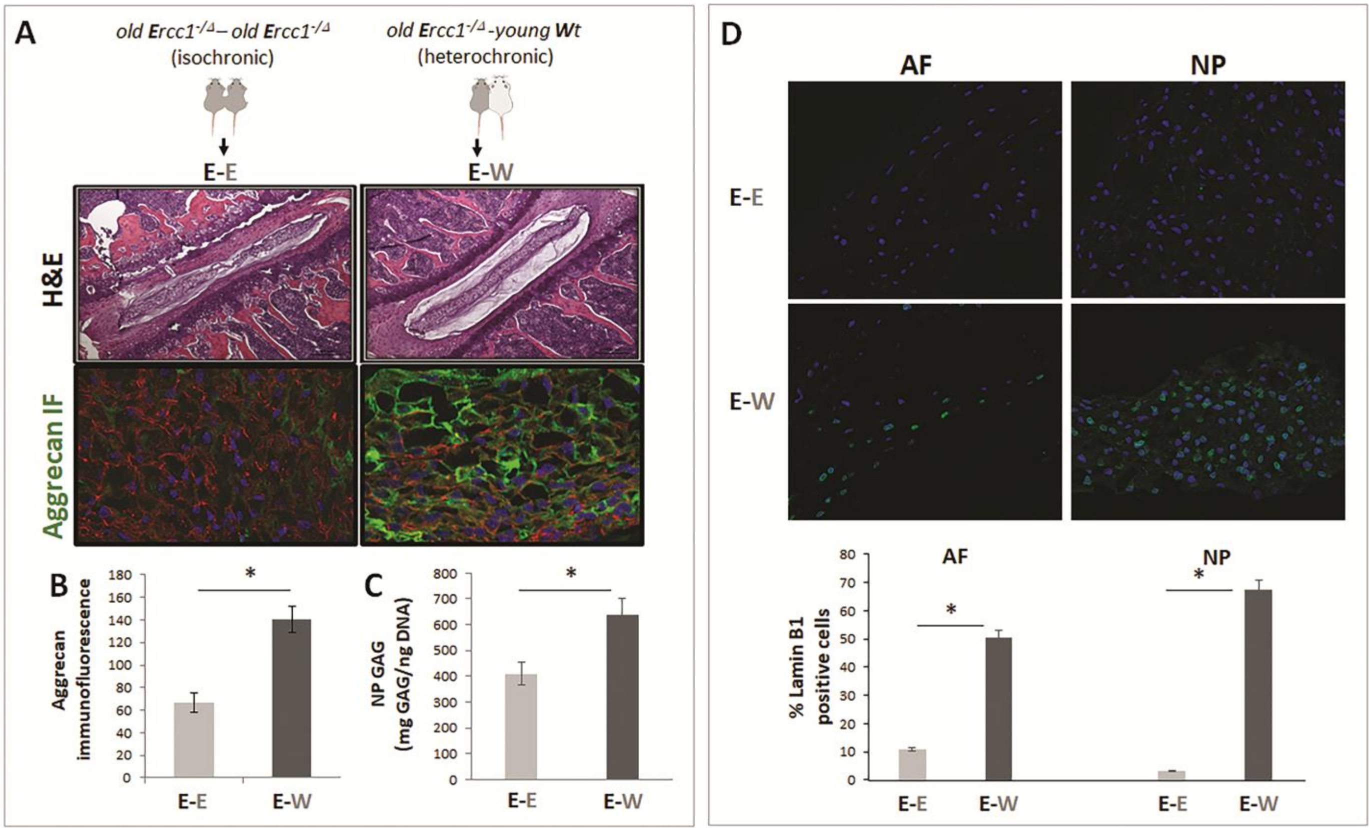

Supplementary Figure 1. Effects of young circulatory factors on disc matrix PG homeostasis in accelerating *Ercc1*$^{-/\Delta}$ mice. (**A**) Discs of *Ercc1*$^{-/\Delta}$ mice from heterochronic parabiosis showed improved histologic features and increased NP aggrecan immunofluorescence compared to those measured in *Ercc1*$^{-/\Delta}$ mice from an isochronic model. Discs of *Ercc1*$^{-/\Delta}$ mice from heterochronic parabiosis showed increased NP aggrecan immunofluorescence (**B**) and GAG content (**C**) compared to those in *Ercc1*$^{-/\Delta}$ mice from an isochronic model. (**D**) Quantitative immunofluorescence of a cellular senescence marker, lamin B, in AF and NP tissues of isochronic and heterochronic *Ercc1*$^{-/\Delta}$ mice. Data shown are mean ± *SD* of 3-5 independent experiments, *$p < 0.05$.

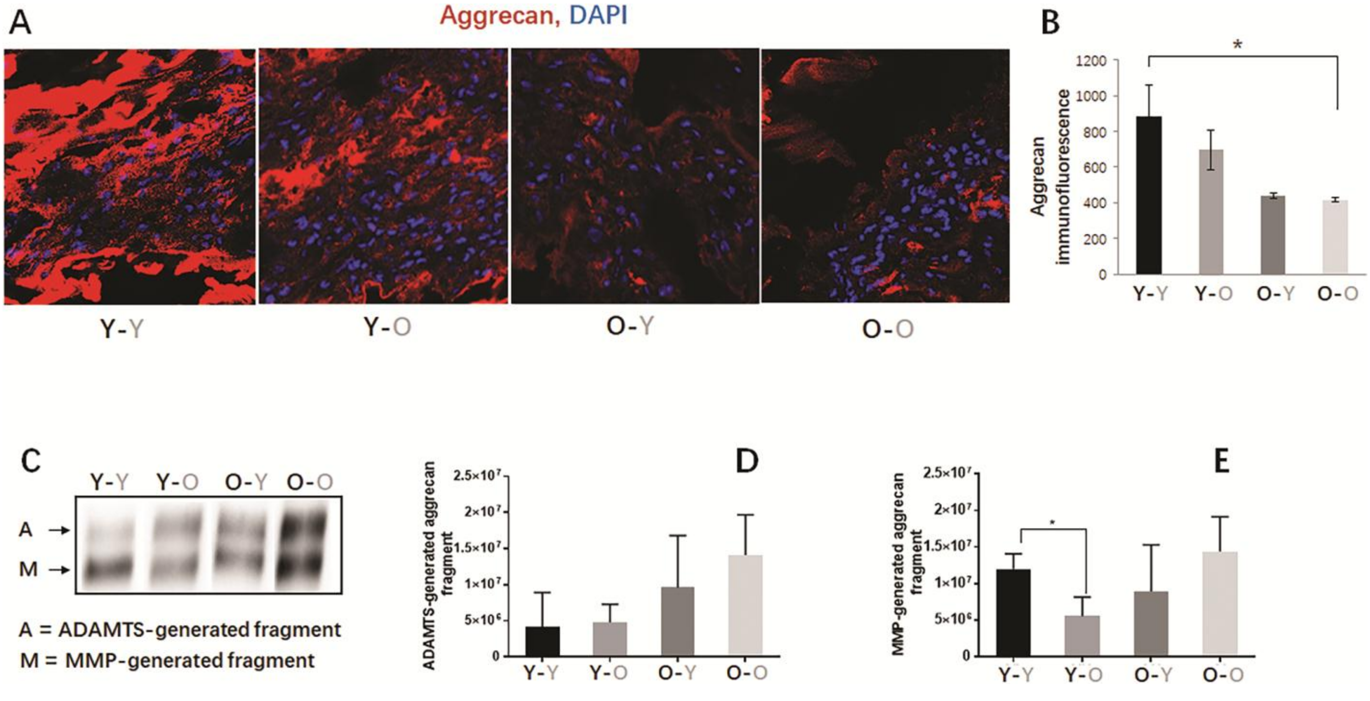

Supplementary Figure 2. Influences of circulatory factors on matrix homeostasis in the intervertebral discs of mice heterochronically-paired for one month. (**A**) Representative images showing the effects of circulatory factors on aggrecan immunofluorescence in the nucleus pulposus of mouse parabionts. (**B**) Quantification of the NP aggrecan immunofluorescence. shown in the bar graph. Data shown are mean ± *SEM* of four independent experiments (n=4 per group), *$p < 0.05$. (**C**) representative immunoblots of MMP- and ADAMTS-mediated cleavage of disc aggrecan of the four different mouse parabionts. Graphs on right are quantification of ADAMTS-generated aggrecan fragment (**D**) and MMP-generated aggrecan fragment (**E**). Data shown are mean ± *SD* of four independent experiments, *$p < 0.05$.

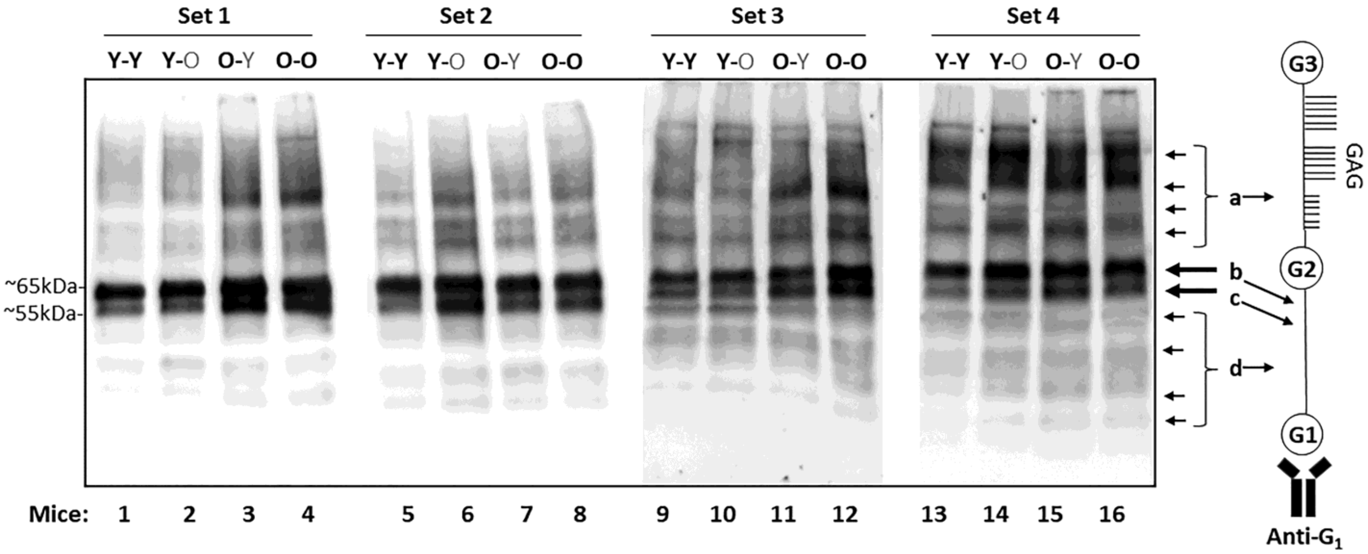

Supplementary Figure 3. Full Western Blots from Figure 2 are shown here. Anti-G1 detected multiple aggrecan fragments derived from cleavage sites between G2 and G3 globular domains (**a**) and between G1 and G2 globular domains (**b**, **c**, **d**). For the sake of clarity, we chose to show only the major proteolytic aggrecan fragments derived from the cleavage sites between G1 and G2 interglobular domains by ADAMTS (**b**) and MMP (**c**) proteases in Figure 2 because these fragments resulted in loss of the entire GAG region from the aggrecan aggregate in disc tissue and are commonly considered most pathological.

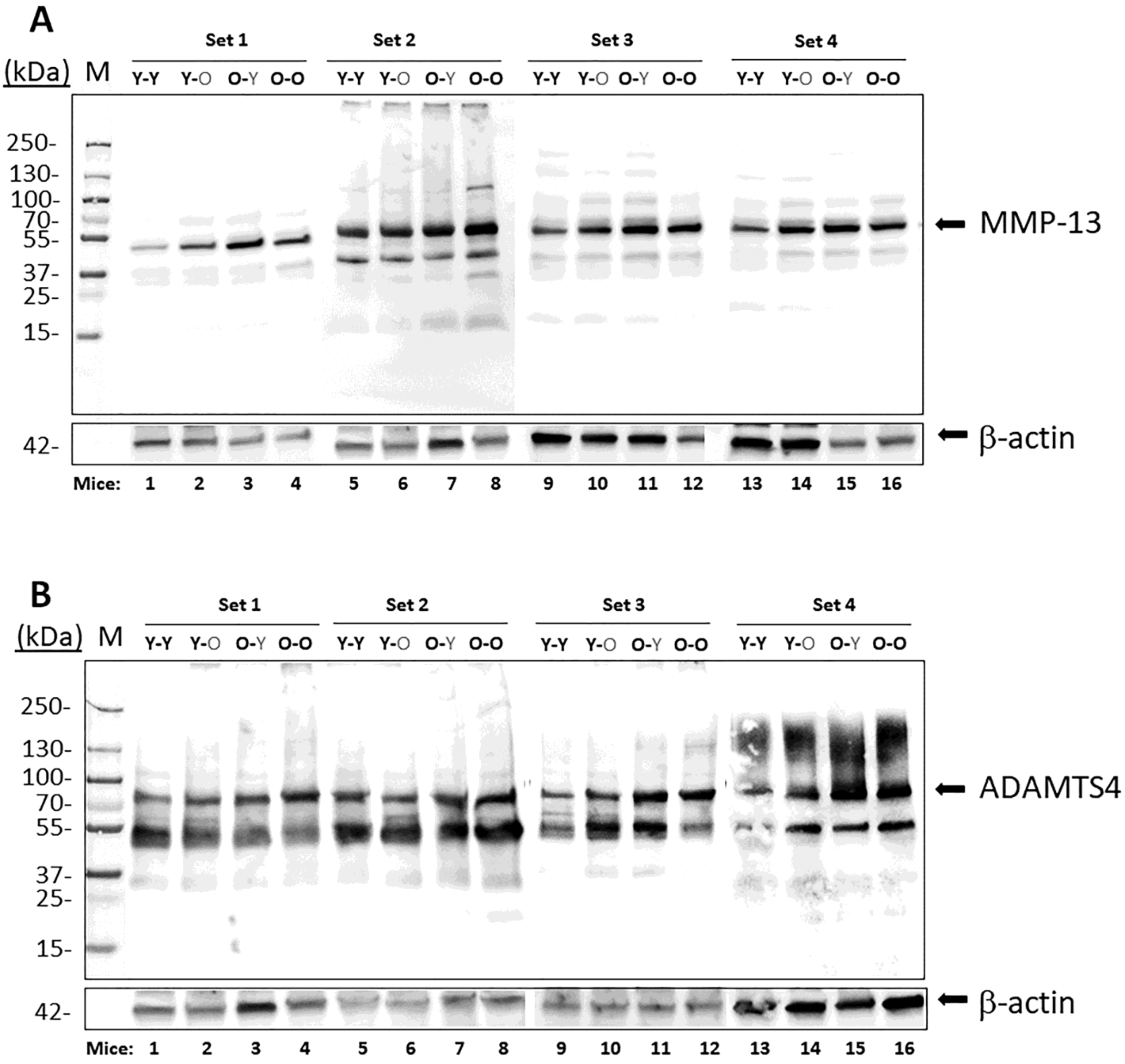

Supplementary Figure 4. Full Western Blots for MMP13 (**A**) and ADAMTS4 (**B**) from which Figure 3 were derived.

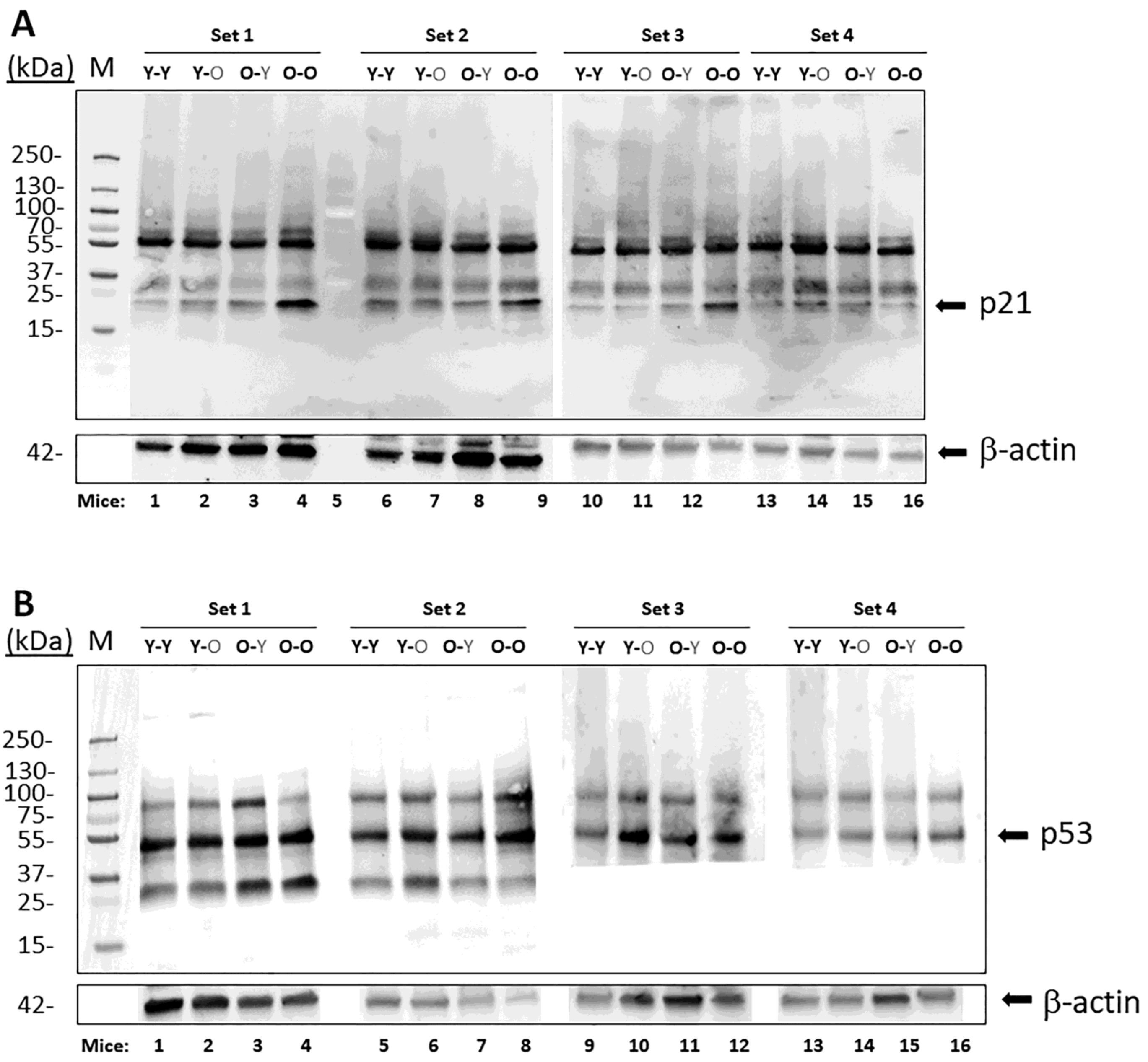

Supplementary Figure 5. Full Western Blots for p21 (**A**) and p53 (**B**) from which Figure 4 were derived.
